# Proposal for subclassification to select patients for hepatectomy with intermediate hepatocellular carcinoma and Child–Pugh A liver function: A double-center study from China

**DOI:** 10.1097/MD.0000000000011800

**Published:** 2018-08-10

**Authors:** Wei Xu, Quan Rao, Yongbo An, Mengyi Li, Gang Xu, Xinting Sang, Xin Lu, Zhongtao Zhang, Yilei Mao

**Affiliations:** a Department of General Surgery, Beijing Friendship Hospital, Capital Medical University, Beijing Key Laboratory of Cancer Invasion and Metastasis Research, National Clinical Research Center for Digestive Diseases; b Department of Liver Surgery, Peking Union Medical College Hospital, Chinese Academy of Medical Sciences and Peking Union Medical College, Beijing, PR China.

**Keywords:** clinical stage, hepatectomy, hepatocellular carcinoma, intermediate stage, prognosis

## Abstract

Increasing evidence has shown that hepatectomy provides a longer overall survival (OS) for patients with hepatocellular carcinoma (HCC) in the intermediate stage. Unfortunately, not all patients benefit from liver resection, even if hepatectomy is feasible. This study aimed to propose a subclassification to select patients for surgical resection.

OS of patients with intermediate-stage HCC who underwent hepatectomy at Beijing Friendship Hospital or Peking Union Medical College Hospital were reviewed. Patients were divided into 2 groups based on the results of survival analysis. The prognosis of these patients was compared with that in those who were treated by trans-arterial chemoembolization (TACE) in each subgroup.

A total of 259 patients with intermediate-stage HCC who were initially treated by hepatectomy were included. Multivariate analysis showed that cumulative tumor size and tumor number independently affected tumor recurrence and survival time of these patients. Patients were then divided into group A (tumor size <11 cm and tumor number < 4; n = 205) and group B (tumor size ≥11 cm and tumor number ≥ 4; n = 54). Multivariate analysis showed that hepatectomy was independently associated with longer OS compared with TACE in patients in group A (hazard ratio = 0.67, 95% confidence interval = 0.49–0.90), but not in group B.

Surgical management of intermediate-stage HCC should be performed with more complexity than current practice. Hepatic resection could be considered as the first-line treatment only for patients with HCC who have a cumulative tumor size of less than 11 cm and <4 tumors.

## 1 Introduction

Hepatocellular carcinoma (HCC) has the fifth highest incidence among types of cancer and has the third highest cancer mortality worldwide.^[1]^ The Barcelona Clinic Liver Cancer (BCLC) staging system is a commonly used staging system accounting for tumor burden, liver function, and other general conditions in HCC.^[2]^ According to the BCLC staging system, curative treatment, including hepatic resection and radio-frequency ablation, are only indicated for patients with HCC in the early stage, while those with the intermediate stage should have palliative treatment.^[3]^ However, increasing evidence has shown that overall survival (OS) of patients with intermediate HCC and Child–Pugh A liver function treated by hepatic resection is longer than that in those after palliative treatment, especially in Asia-Pacific studies.^[4–11]^ Hepatic resection may result in a survival benefit over trans-arterial chemoembolization (TACE) and should be considered as the first-line treatment.

Not all patients with intermediate-stage HCC benefit from liver resection, even if hepatectomy is feasible. The prognosis of patients with HCC in the intermediate stage is still poor after surgical treatment, with many dying within 1 year, or even in the perioperative period.^[12]^ We speculate that only some of these patients would have improved survival rates with hepatectomy because intermediate-stage HCC is a heterogeneous category. Subclassifications of stage B patients based on outcomes of HCC have been reported; and correlations between these subclassifications and effectiveness of TACE treatment have been demonstrated.^[13,14]^ However, little information is currently available regarding which patients with intermediate-stage HCC should consider hepatectomy as the first-line treatment, especially for those with large multifocal tumors treated by liver resection.

More information on intermediate-stage HCC is necessary because specific analyses of surgical patient subgroups to detect prognostic factors with liver resection may further predict patients’ outcomes. Therefore, we investigated B stage subclassifications to facilitate more appropriate management of these patients. In the present study, we conducted a double center, retrospective study from the National Clinical Research Centre for Digestive Disease in China (Beijing Friendship Hospital) and Department of Liver Surgery (Peking Union Medical College Hospital). We analyzed information to identify patients with intermediate-stage HCC who may have potential survival benefit from hepatectomy.

## 2 Methods

### 2.1 Patients

Patients with BCLC stage B HCC who underwent curative resection or TACE between January 2000 and January 2012 at Beijing Friendship Hospital or Peking Union Medical College Hospital were identified from databases. Patients with intermediate-stage cancer who met the diagnostic criteria and treatment methods were included. However, those with other initial treatments (except for hepatic resection or TACE) who suffered from previous or simultaneous multiple primary malignancies, or had vague clinical-pathological parameters (e.g., tumor size) were excluded. The study protocol was approved by the institutional review board or ethics committee of each participating institution.

The 2 hospitals that participated in the present study serve as the National Clinical Research Centre for Digestive Disease or Liver Disease centers in China. Each hospital employs specialists in hepatology, radiology, and surgery for diagnosis and treatment of HCC. The quality of clinical practice and records were assessed by 2 statisticians.

### 2.2 Diagnostic criteria and treatment methods

Diagnosis of HCC was based on abdominal ultrasonography plus dynamic computed tomography (CT), magnetic resonance imaging (MRI), or both, as recommended by the *Updated Standards for the Diagnosis and Treatment of Primary Liver Cancer*.^[15]^ Patients who were treated by surgical resection were confirmed by pathology. BCLC stage B HCC was defined as HCC in the intermediate stage. These patients had Child–Pugh grade A or B liver function, large, multifocal tumors (cumulative tumor size >5 cm or >3 tumor nodules), and the absence of cancer-related symptoms, macrovascular invasion, or extra-hepatic spread.^[16]^

The preoperative conditions of patients who were treated by hepatectomy were reassessed to ensure that they conformed to the surgical resection criteria outlined in the *Updated Standards for the Diagnosis and Treatment of Primary Liver Cancer*. These criteria include a normal general condition with no obvious pathological changes to the heart, lungs, kidney, and other important organs, and normal or reserved liver function with short-term therapy for protecting the liver.

All surgical planning and operations were performed with similar surgical techniques as follows. The resection area was determined with the aim of removing its deepest portion, combining minimal parenchymal sacrifice and the flattest cut surface. Hilar dissection with the intent to perform systemic lymphadenectomy was not routinely performed, except in patients who were suspected as having metastatic lymph nodes on preoperative magnetic resonance imaging or computed tomography. Parenchymal transection was performed by the Cavitron ultrasonic aspiration system or Ligasure as the operating surgeon's preference. The hilar pedicle was encircled by tourniquets, and transient hepatic inflow occlusion during parenchymal transection was obtained by Pringle's maneuver. Upon conclusion of liver transection, the cut surface of the liver was routinely monitored for 20 minutes, and any sites of bleeding or bile leakage were well controlled.

The records of patients who were treated by TACE were also re-evaluated. The conditions of these patients, such as pretreatment liver function, renal function, and coagulation function, were confirmed using the TACE criteria outlined in the *Updated Standards for the Diagnosis and Treatment of Primary Liver Cancer*.^[15]^ Similar planning and techniques were performed and reassessed.

### 2.3 Clinical and pathological features

Clinical data were collected, including those for age, sex, and laboratory test results, including levels of alanine aminotransferase (ALT), aspartate aminotransferase (AST), total bilirubin, and albumin, prothrombin time (PT), hemoglobin, serum hepatitis B virus (HBV) surface antigen (HBsAg) levels, hepatitis C virus (HCV) antibody levels, and serum alpha-fetoprotein (AFP) levels. Information regarding tumor number, cumulative tumor size, and extra-hepatic spread was recorded based on CT or MRI. Liver function was assessed by the Child–Pugh scoring system. Pathological features, such as tumor differentiation, were required in the resection group and were graded by the Edmondson grading system.^[17]^

### 2.4 Surveillance for tumor recurrence and follow-up

All of the patients were regularly followed up by the outpatient department. The patients were prospectively monitored for recurrence with a standard protocol that included measurement of serum AFP levels, an ultrasound examination, and a contrast CT or MRI examination. Patients were followed up every 3 months during the first postoperative year and at least every 6 months thereafter. Abdominal CT or MRI scans were performed every 6 months. Recurrence was diagnosed based on the typical imaging appearance on CT or MRI. A positron emission tomography scan was suggested in patients who underwent surgery unless new suspicious lesions were detected by CT or MRI. Further appropriate treatments could be used for patients who were diagnosed with HCC recurrence. OS was defined as the interval between surgery and death or the last date of follow-up. Disease-free survival (DFS) was calculated from the date of resection to the date when tumor recurrence was diagnosed. If recurrence was not diagnosed at the time of study, the cases were censored on the date of death or the last date of follow-up.

### 2.5 Statistical analysis

Clinical and pathological factors of the groups were compared using Fisher's exact test or Pearson's χ^2^-test, as appropriate. The survival rate was calculated using the Kaplan–Meier method. Cox regression analysis was performed to identify independent risk factors with the hazard ratio (HR) and 95% confidence interval (CI). A value of *P* < .05 was considered statistically significant. Outcome-based threshold optimization was assessed by using *X*-title software.^[18]^ Data analysis was performed using SPSS 19.0 software.

## 3 Results

### 3.1 Patients

A total of 631 patients with BCLC stage B HCC were included. Among them, 259 patients were treated with resection and 372 with TACE. The baseline demographic data and tumor characteristics are shown in Table 1. The majority of the characteristics that were examined in the resection and TACE groups were comparable, including age, sex, and preoperative liver function. For liver function, 100% and 98.9% patients scored Child–Pugh Class A, respectively. More patients suffered from 4 or more tumors in the TACE group than in resection group, and both groups had a mean cumulative tumor size >8 cm.

**Table 1:**
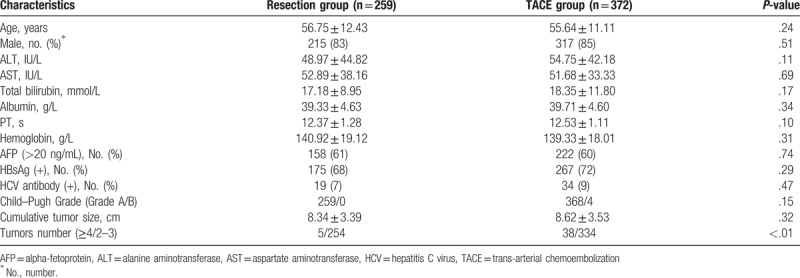
Clinical characteristics of patients with intermediate-stage hepatocellular carcinoma.

### 3.2 Survival analysis of patients with BCLC stage B HCC

**Figure 1:**
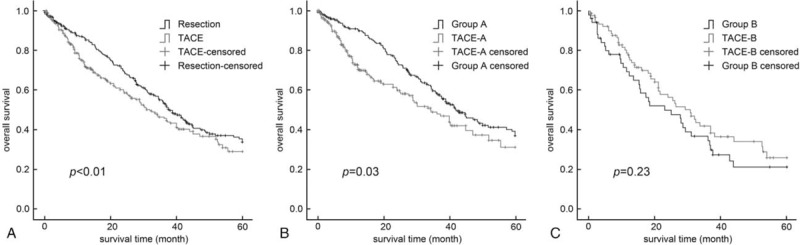
(A) Survival analysis of patients with intermediate-stage HCC who underwent resection (H, *P = *.03). (B) Survival analysis in group A vs the TACE-A subgroup (*P*<.01). (C) Survival analysis in group B vs the TACE-B subgroup (*P = *.23). HCC = hepatocellular carcinoma, TACE = trans-arterial chemoembolization.

The final follow-up for all patients was February 2017. The median follow-up time of patients with BCLC stage B HCC was 37.6 months (range, 0.3–60 months). For patients with BCLC stage B HCC who were treated by resection, the 1-, 3-, and 5-year DFS rates were 77.1%, 46.2%, and 27.3%, respectively, and OS rates were 86.5%, 53.8%, and 33.8%, respectively. The perioperative death rate of these patients was 1.9% (5/259). The 1-, 3-, and 5-year OS rates (73.9%, 45.7%, and 28.9%, respectively) for patients with TACE were significantly lower than those treated by liver resection (*P = *.03, Fig. 1A).

**Figure 2:**
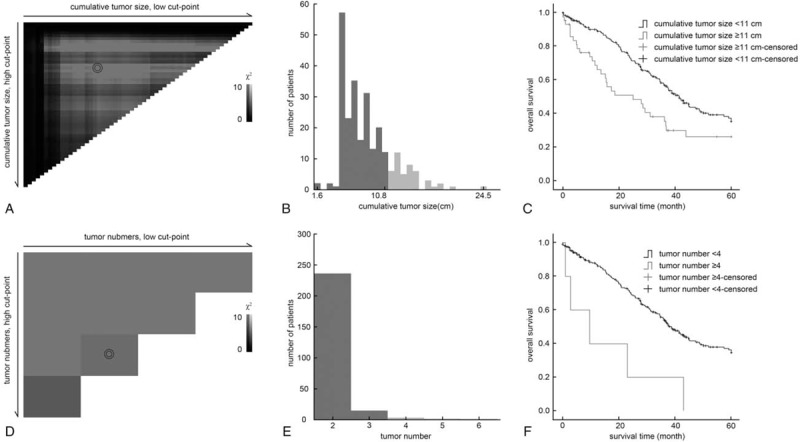
Optimal threshold selection of cumulative tumor size and tumor number based on *X*-tile software. (A) Tumor size was divided at the optimal threshold, as defined by the most significant (brightest pixel) point on the plot. Diffuse bright pixels indicate a continuous indirect association between increasing tumor size and good prognosis. (B) Relationship between the number of patients and deviation in tumor size. (C) Survival analysis based on deviation in tumor size. (D) The tumor number divided at the optimal threshold was 4. (E) Relationship between the number of patients and tumor number. (F) Survival analysis based on deviation of tumor number.

Univariate analysis showed that cumulative tumor size, tumor number, Edmondson grade, AFP, HBsAg, AST, and PT were prognostic factors for DFS or OS of patients with BCLC stage B HCC. Multivariate analysis showed that a tumor number of ≥4 and cumulative tumor size ≥11 cm were independent risk factors for tumor recurrence and poor prognosis of these patients (Table 2). Moreover, 11 cm was selected as the threshold for the cumulative tumor size (Fig. 2A–C) and 4 was selected as the threshold for the number of tumors (Fig. 2D–F) based on the results of survival-associated analysis by *X*-title software.

**Table 2:**
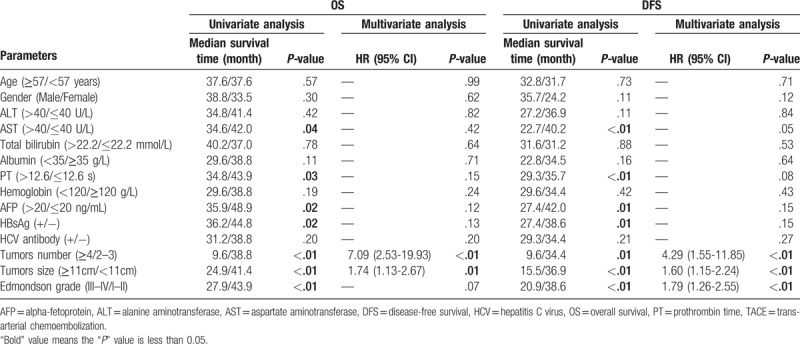
Univariate and multivariate analyses of risk factors related to overall survival of patients with intermediate-stage hepatocellular carcinoma treated by resection.

### 3.3 Group classification

Based on the results of multivariate analysis, cumulative tumor size and tumor number were selected for group classification. Patients with intermediate-stage HCC who had surgical treatment were divided into group A (cumulative tumor size <11 cm and tumor number <4; n = 205) and group B (cumulative tumor size ≥11 cm or tumor number ≥4; n = 54). Patients with HCC in the TACE group were also divided into subgroups by the criteria mentioned above (TACE-A, n = 279; TACE-B, n = 93; Table 3).

**Table 3:**
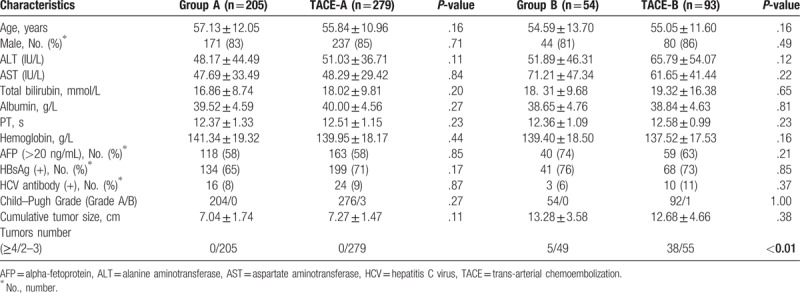
Clinical characteristics of patients with intermediate-stage hepatocellular carcinoma.

### 3.4 Clinical characteristics and OS of the subgroups

Baseline demographic data and tumor characteristics of patients who received hepatic resection were re-evaluated in each subgroup and compared with those of patients who were treated by TACE (Table 3). For patients in group A, the baseline demographic data and tumor characteristics were comparable with patients in the TACE-A subgroup. The 1-, 3-, and 5-year cumulative survival rates in group A patients were 90.4%, 58.3%, and 37.1%, respectively, and their perioperative death rate was 1.5% (3/205). For patients in the TACE-A subgroup, the 1-, 3-, and 5-year cumulative survival rates were 72.5%, 47.8%, and 31.2%, respectively (Fig. 1B). Univariate analysis showed that AST levels, albumin levels, PT, AFP levels, tumor size, and treatment methods significantly affected the OS of patients. Multivariate analysis showed that hepatectomy resulted in longer OS in patients in group A compared with TACE independently (HR = 0.67, 95% CI = 0.49–0.90) (Table 4).

**Table 4:**
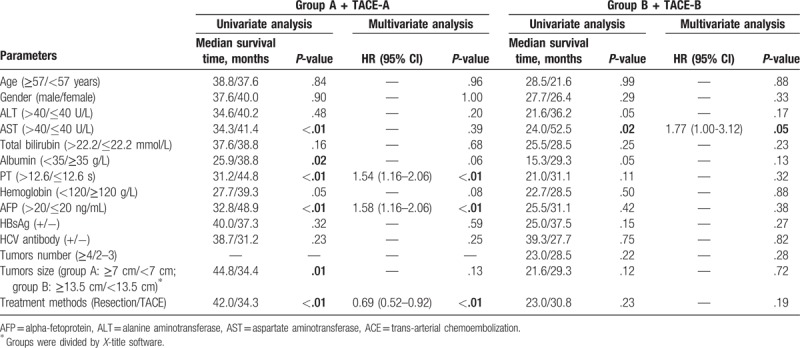
Univariate and multivariate analyses of risk factors related to overall survival of patients with intermediate-stage hepatocellular carcinoma in subgroups.

For patients in group B, most of the clinical characteristics were comparable with those in the TACE-B subgroup, except for the number of tumors. Interestingly, although patients in the TACE-B subgroup suffered from more tumor lesions, their 1-, 3-, and 5-year cumulative survival rates were comparable with those in group B patients (77.9%, 38.5%, and 26.0% vs 67.2%, 34.6%, and 21.4%, respectively, *P = *.23, Fig. 1C). Furthermore, the perioperative death rate of group B patients was 3.7% (2/54). Univariate analysis showed that only AST was associated with prognosis of patients with HCC. Multivariate analysis showed that surgical resection did not lead to a survival benefit in patients in group B (Table 4).

## 4 Discussion

Increasing evidence has shown that liver resection provides a longer OS for patients with BCLC stage B than palliative treatments. Hepatic resection is considered a safe and effective treatment for large multinodular HCC. When technically feasible and clinically appropriate, liver resection should be performed for this condition.^[19]^ However, recent studies have shown that not all patients with BCLC stage B HCC receive a survival benefit from hepatectomy. Additionally, liver resection is even regarded as a futile procedure for those with unfavorable surgical outcomes, such as perioperative death or death within 1 year of surgery because of tumor recurrence.^[20]^ Our previous research showed that nearly 20% of patients with BCLC stage B HCC died within 1 year, even if they received surgical treatment, rigorous postoperative monitoring, and appropriate treatment for recurrent tumors.^[12]^ Therefore, a thorough selection process is essential to ensure that only patients who may receive a potential survival benefit may proceed to liver resection.

In the present double-center study, a large number of patients with BCLC stage B HCC with preoperative liver function of Child–Pugh class A were included. Multivariate analysis showed that cumulative tumor size and tumor number were independently associated with surgical outcome for HCC. Based on *X*-title software analysis, the optimized threshold for cumulative tumor size was 11 cm and that for the number of tumors was 4. Patients with large multinodular HCC within these 2 cut-off values had a 1-year postoperative cumulative survival rate of 90.4% and a perioperative death rate of 1.5%. Further, univariate and multivariate analyses showed that these patients received more prognostic improvement with resection rather than TACE. In contrast, for patients with a cumulative tumor size ≥11 cm or ≥4 tumors, resection was not an independent protective risk factor for survival in multivariate Cox regression models.

The present study proposed a selection criterion of a cumulative tumor size less than 11 cm for patients with BCLC stage B HCC who were preparing to receive curative intent surgery. This criterion radically differs from recent findings by Vitale et al.^[9]^ They argued that tumor size may not have an negative effect on the benefit of resection in intermediate stage HCC, suggesting that aggressive treatments should be considered, when clinically applicable, to achieve the maximum survival benefit. In contrast, cumulative tumor size independently affected OS of patients with BCLC stage B HCC in some series,^[13,14,21–24]^ but there has been no consensus on which criteria should be used.

Bolondi et al proposed a subclassification of intermediate-stage HCC.^[13]^ This classification incorporates the “beyond Milan and up-to-7 tumors” criteria, a concept that combines the size and number of tumors.^[21]^ In Bolondi's substaging system, resection was only considered as an alternative treatment method of TACE for HCC within the “up-to-7 criteria.” This system is reportedly useful in the stratification of patients with intermediate-stage HCC because prognosis worsens as substaging progresses,^[22]^ but the recommended treatment methods remain controversial. Ciria *et al.* showed that patients with HCC within the up-to-7 criteria should consider resection as the first-line treatment ^[23]^ because the OS rate with resection was significantly higher than that with TACE. Even with HCC tumors >7 cm, resection may also be considered if anatomical and pathological acceptable criteria are met. Additionally, in a report by Weinmann et al,^[14]^ no prognostic meaning was identified for Bolondi's subclassification.

Arizumi et al^[24]^ modified criteria for Bolondi's substaging system and recommended resection or even ablation for patients who met the up-to-7 criteria with well-preserved liver function. Although the Kinki criteria were suitable for subclassification of patients with intermediate stage HCC in a study by Arizumi *et al.*^[24]^, more than 55% patients were classified as B2 stage and lost their chance of receiving surgery. Additionally, the effect of their treatment method was not shown in their series. Another criterion described as “within up-to-10” was proposed in some series.^[10]^ Although the effect of cumulative tumor size was shown in these studies, the threshold was determined based on clinical practice. In the present study, more than half of the patients treated by resection (54.1%, 140/259) were beyond the up-to-7 criteria. Therefore, the Kinki criteria may be too narrow, and do not fit our patient series. We developed a more expanded criteria as “up-to-11,” for cut-off values for continuous clinical factors as determined by *X*-title software statistical analysis. The survival benefits achieved by liver resection were shown in multivariate analysis.

In our study, we also added “tumor number <4’ to clarify the role of hepatic resection in patients with intermediate HCC. This criterion is in accordance with the Evidence-Based Clinical Practice Guidelines in Japan and KLCSG-NCC Korea Practice Guideline for the Management of Hepatocellular Carcinoma.^[25,26]^ Interestingly, the number of surgical procedures sharply decreased with a tumor number of >3 in our series, possibly because of anatomically unacceptable or insufficient remaining liver volume after resection. In the resection group, more than 90% (239/259) of patients in BCLC stage B suffered from HCC with 2 lesions, while only 2% (5/259) had more than three lesions. Furthermore, a tumor number of <4 may reflect anatomical or clinical possibility of surgery. Multiple tumors in the resection group tended to be in adjacent subsections or limited to one half of the liver, so that residual liver tissue was retained to a large extent after hepatectomy.

Liver function is a well-known prognostic factor in patients with HCC undergoing liver resection.^[27,28]^ In many series, Child–Pugh A class liver function was a necessary condition for not only resection, but also TACE.^[20,23]^ In the present study, all patients with BCLC stage B HCC who received hepatectomy had preoperative liver function in Child–Pugh A class, which fits the *Updated Standards for the Diagnosis and Treatment of Primary Liver Cancer* in China.^[15]^ For patients with Child–Pugh class B with a major tumor burden, liver resection may increase the risk of excessive liver damage owing to the need for massive hepatectomy.

We propose a subclassification for selecting candidate patients for hepatectomy with Child–Pugh class A liver function as follows: tumor burden within the up-to-11 criteria and a tumor number of <4. Patients may receive more prognostic benefit from liver resection than from TACE. Therefore, resection should be considered as the first-line treatment for these patients if surgery is clinically and anatomically applicable. The OS of patients exceeding the up-to-11 criteria or a tumor number of ≥4 was not as good as that for up-to-11 criteria or a tumor number of <4. However, TACE may still be recommended according to the BCLC staging system. Further studies are warranted to determine the optimal treatment methods for these patients.

This study has some limitations that are inherent in retrospective studies. These limitations include the following: observational data collected at a specific point; missing data because of patients lost to follow-up; difficulty in obtaining some pathological data, such as etiologies of liver disease besides HBV or HCV; cirrhosis and its severity; and selection bias. In the absence of adequately powered randomized clinical trials, propensity score analysis should have been used in this study to most closely simulate a randomized clinical trial. However, our study was limited by our relatively small sample size, making a truly matched study unfeasible. Some important therapeutic methods, such as hepatic resection after TACE or associating liver partition and portal vein ligation for staged hepatectomy, were not included in the present study. Although 2 hospitals participated in the present study, to ensure our results are applicable beyond China, a larger series of patients from a multi-geographic patient base is required in the future.

## Acknowledgments

We thank Ellen Knapp, PhD, from Liwen Bianji, Edanz Group China (www.liwenbianji.cn/ac), for editing the English text of a draft of this manuscript.

## Author contributions

**Conceptualization:** Quan Rao, Yongbo An, Mengyi Li, Gang Xu.

**Data curation:** Wei Xu, Quan Rao, Yongbo An, Mengyi Li, Gang Xu.

**Formal analysis:** Wei Xu, Quan Rao, Yongbo An, Mengyi Li.

**Funding acquisition:** Wei Xu, Gang Xu, Xinting Sang, Xin Lu.

**Investigation:** Quan Rao, Yongbo An, Mengyi Li, Gang Xu, Xinting Sang, Xin Lu.

**Methodology:** Quan Rao, Yongbo An, Mengyi Li, Gang Xu, Xinting Sang, Xin Lu.

**Project administration:** Wei Xu, Gang Xu, Xinting Sang, Xin Lu.

**Resources:** Mengyi Li, Xinting Sang, Xin Lu.

**Software:** Xinting Sang, Xin Lu.

**Supervision:** Xinting Sang, Xin Lu, Zhongtao Zhang.

**Validation:** Zhongtao Zhang, Yilei Mao.

**Visualization:** Wei Xu, Zhongtao Zhang, Yilei Mao.

**Writing – original draft:** Wei Xu, Yilei Mao.

**Writing – review & editing:** Wei Xu, Zhongtao Zhang, Yilei Mao.

## References

[1] Xu W Xu H Yang H Continuous Pringle maneuver does not affect outcomes of patients with hepatocellular carcinoma after curative resection. Asia Pac J Clin Oncol 2017;13:e321–30. 27519165 10.1111/ajco.12585

[2] Díaz-González Á Reig M Bruix J Treatment of hepatocellular carcinoma. Dig Dis 2016;34:597–602. 27332893 10.1159/000445275

[3] Bruix J Sherman M American Association for the Study of Liver Diseases. Management of hepatocellular carcinoma: an update. Hepatology 2011;53:1020–2. 21374666 10.1002/hep.24199 PMC3084991

[4] Qi X Wang D Su C Hepatic resection versus trans-arterial chemoembolization for the initial treatment of hepatocellular carcinoma: a systematic review and meta-analysis. Oncotarget 2015;6:18715–33. 26243835 10.18632/oncotarget.4134 PMC4621923

[5] Liu W Zhou JG Sun Y Hepatic resection improved the long-term survival of patients with BCLC stage B hepatocellular carcinoma in Asia: a systematic review and meta-analysis. J Gastrointest Surg 2015;19:1271–80. 25943910 10.1007/s11605-015-2811-6

[6] Tada T Kumada T Toyoda H Role of hepatic resection in patients with intermediate-stage hepatocellular carcinoma: A multicenter study from Japan. Cancer Sci 2017;108:1414–20. 28406546 10.1111/cas.13257 PMC5497930

[7] Kokudo T Hasegawa K Matsuyama Y Survival benefit of liver resection for hepatocellular carcinoma associated with portal vein invasion. J Hepatol 2016;65:938–43. 27266618 10.1016/j.jhep.2016.05.044

[8] Bhandare MS Patkar S Shetty N Liver resection for HCC outside the BCLC criteria. Langenbecks Arch Surg 2017;4. 10.1007/s00423-017-1640-3 29199380

[9] Vitale A Burra P Frigo AC Survival benefit of liver resection for patients with hepatocellular carcinoma across different Barcelona Clinic Liver Cancer stages: a multicentre study. J Hepatol 2015;62:617–24. 25450706 10.1016/j.jhep.2014.10.037

[10] Jianyong L Lunan Y Wentao W Barcelona clinic liver cancer stage B hepatocellular carcinoma: transarterial chemoembolization or hepatic resection? Medicine (Baltimore) 2014;93:e180. 25474433 10.1097/MD.0000000000000180 PMC4616388

[11] Zhong JH Xiang BD Gong WF Comparison of long-term survival of patients with BCLC stage B hepatocellular carcinoma after liver resection or transarterial chemoembolization. PLoS One 2013;8:e68193. 23874536 10.1371/journal.pone.0068193 PMC3706592

[12] Wei Xu Rui Guo Gang Xu Management of intrahepatic recurrence after resection for hepatocellular carcinoma exceeding the Barcelona Clinic Liver Cancer criteria. Oncotarget 2017;8:110406–14. 29299157 10.18632/oncotarget.22779 PMC5746392

[13] Bolondi L Burroughs A Dufour JF Heterogeneity of patients with intermediate (BCLC B) hepatocellular carcinoma: proposal for a subclassification to facilitate treatment decisions. Semin Liver Dis 2012;32:348–59. 23397536 10.1055/s-0032-1329906

[14] Weinmann A Koch S Sprinzl M Survival analysis of proposed BCLC-B subgroups in hepatocellular carcinoma patients. Liver Int 2015;35:591–600. 25290314 10.1111/liv.12696

[15] Ministry of Health of the People's Republic of China. Updated standards for the diagnosis and treatment of primary liver cancer. Zhonghua Gan Zang Bing Za Zhi 2012;20:419–26. article in Chinese. 23230592

[16] Bruix J Reig M Sherman M Evidence-based diagnosis, staging, and treatment of patients with hepatocellular carcinoma. Gastroenterology 2016;150:835–53. 26795574 10.1053/j.gastro.2015.12.041

[17] Edmondson HA Steiner PE Primary carcinoma of the liver: a study of 100 cases among 48,900 necropsies. Cancer 1954;7:462–503. 13160935 10.1002/1097-0142(195405)7:3<462::aid-cncr2820070308>3.0.co;2-e

[18] Camp RL Dolled-Filhart M Rimm DL X-tile: a new bio-informatics tool for biomarker assessment and outcome-based cut-point optimization. Clin Cancer Res 2004;10:7252–9. 15534099 10.1158/1078-0432.CCR-04-0713

[19] Zhong JH Rodríguez AC Ke Y Hepatic resection as a safe and effective treatment for hepatocellular carcinoma involving a single large tumor, multiple tumors, or macrovascular invasion. Medicine (Baltimore) 2015;94:e396. 25621684 10.1097/MD.0000000000000396 PMC4602643

[20] Li C Shen JY Zhang XY Predictors of futile liver resection for patients with Barcelona Clinic Liver Cancer stage B/C hepatocellular carcinoma. J Gastrointest Surg 2018;22:496–502. 29119530 10.1007/s11605-017-3632-6

[21] Mazzaferro V Llovet JM Miceli R Predicting survival after liver transplantation in patients with hepatocellular carcinoma beyond the Milan criteria: a retrospective, exploratory analysis. Lancet Oncol 2009;10:35–43. 19058754 10.1016/S1470-2045(08)70284-5

[22] Scaffaro LA Stella SF Alvares-Da-Silva MR Survival rates according to Barcelona clinic liver cancer sub-staging system after transarterial embolization for intermediate hepatocellular carcinoma. World J Hepatol 2015;7:628–32. 25848487 10.4254/wjh.v7.i3.628 PMC4381186

[23] Ciria R López-Cillero P Gallardo AB Optimizing the management of patients with BCLC stage-B hepatocellular carcinoma: Modern surgical resection as a feasible alternative to transarterial chemoemolization. Eur J Surg Oncol 2015;41:1153–61. 26118317 10.1016/j.ejso.2015.05.023

[24] Arizumi T Ueshima K Iwanishi M Validation of a modified substaging system (Kinki criteria) for patients with intermediate-stage hepatocellular carcinoma. Oncology 2015;89:47–52. 10.1159/000440631 26584036

[25] Kokudo N Hasegawa K Akahane M Evidence-based clinical practice guidelines for hepatocellular carcinoma: the Japan Society of Hepatology 2013 update (3rd JSHHCC Guidelines). Hepatol Res 2015;45:123–7. 10.1111/hepr.12464 25625806

[26] Korean Liver Cancer Study Group (KLCSG); National Cancer Center, Korea (NCC). 2014 KLCSG-NCC Korea Practice Guideline for the Management of Hepatocellular Carcinoma. Gut Liver 2015;9:267–317. 25918260 10.5009/gnl14460 PMC4413964

[27] Forner A Reig ME de Lope CR Current strategy for staging and treatment: the BCLC update and future prospects. Semin Liver Dis 2010;30:61–74. 20175034 10.1055/s-0030-1247133

[28] Zaydfudim VM Chapman WC Nagorney DM Challenges in patient selection for liver resection or transplantation in patients with hepatocellular carcinoma beyond Milan criteria. Hepatobiliary Surg Nutr 2017;6:287–9. 28848757 10.21037/hbsn.2017.07.04 PMC5554769

